# Neurexin dysfunction in neurodevelopmental and neuropsychiatric disorders: a PRIMSA-based systematic review through iPSC and animal models

**DOI:** 10.3389/fnbeh.2024.1297374

**Published:** 2024-02-06

**Authors:** Dan Shan, Yuming Song, Yanyi Zhang, Cheong Wong Ho, Wenxin Xia, Zhi Li, Fenfen Ge, Qifeng Ou, Zijie Dai, Zhihao Dai

**Affiliations:** ^1^Department of Biobehavioral Sciences, Columbia University, New York, NY, United States; ^2^Faculty of Health and Medicine, Lancaster University, Lancaster, United Kingdom; ^3^School of Medical Imaging, Hebei Medical University, Shijiazhuang, China; ^4^School of Medicine, Shanghai Jiao Tong University, Shanghai, China; ^5^School of Medicine, University of Galway, Galway, Ireland; ^6^College of Health, Medicine and Wellbeing, University of Newcastle, Newcastle, NSW, Australia; ^7^Faculty of Medicine, University of Iceland, Reykjavík, Iceland; ^8^Division of Biosciences, Faculty of Life Sciences, University College London, London, United Kingdom; ^9^School of Medicine, Royal College of Surgeons in Ireland, Dublin, Ireland

**Keywords:** animal models, human induced pluripotent stem cells, disease modeling, neurexins, neuropsychiatric diseases

## Abstract

**Background:**

Neurexins, essential synaptic proteins, are linked to neurodevelopmental and neuropsychiatric disorders like autism spectrum disorder (ASD) and schizophrenia.

**Objective:**

Through this systematic review, we aimed to shed light on the relationship between neurexin dysfunction and its implications in neurodevelopmental and neuropsychiatric manifestations. Both animal and human-induced pluripotent stem cell (hiPSC) models served as our primary investigative platforms.

**Methods:**

Utilizing the PRISMA 2020 guidelines, our search strategy involved scouring articles from the PubMed and Google Scholar databases covering a span of two decades (2003–2023). Of the initial collection, 27 rigorously evaluated studies formed the essence of our review.

**Results:**

Our review suggested the significant ties between neurexin anomalies and neurodevelopmental and neuropsychiatric outcomes, most notably ASD. Rodent-based investigations delineated pronounced ASD-associated behaviors, and hiPSC models derived from ASD-diagnosed patients revealed the disruptions in calcium dynamics and synaptic activities. Additionally, our review underlined the integral role of specific neurexin variants, primarily NRXN1, in the pathology of schizophrenia. It was also evident from our observation that neurexin malfunctions were implicated in a broader array of these disorders, including ADHD, intellectual challenges, and seizure disorders.

**Conclusion:**

This review accentuates the cardinal role neurexins play in the pathological process of neurodevelopmental and neuropsychiatric disorders. The findings underscore a critical need for standardized methodologies in developing animal and hiPSC models for future studies, aiming to minimize heterogeneity. Moreover, we highlight the need to expand research into less studied neurexin variants (i.e., NRXN2 and NRXN3), broadening the scope of our understanding in this field. Our observation also projects hiPSC models as potent tools for bridging research gaps, promoting translational research, and fostering the development of patient-specific therapeutic interventions.

## Introduction

Neurexins, primarily transcribed from three genes (in animals: Nrxn1, Nrxn2, Nrxn3; in humans: NRXN1, NRXN2, NRXN3) (Khoja et al., 2023), are pivotal presynaptic adhesion proteins within the nervous system. These proteins play critical roles in synapse formation and function, manifesting in two primary forms: the longer α-neurexins and the shorter β-neurexins (Reissner et al., 2013; Zhang et al., 2022; Trotter et al., 2023). α-neurexins, characterized by six large extracellular laminin/neurexin/sex hormone-binding (LNS) globulin domains and three interspersed epidermal growth factor (EGF)-like regions; along with β-neurexins, featuring only the sixth LNS domain and absent EGF-like regions, both play a pivotal role in synaptic function (Cuttler et al., 2021). These proteins, expressed at both excitatory and inhibitory synapses (Reissner et al., 2013), contribute to neurotransmission, synaptic plasticity, and neuronal development (Trotter et al., 2023). Their structures, particularly in the neurexin 1 protein, have been elucidated in various species, though human-specific structural data remains elusive (Cuttler et al., 2021).

The intricacies of neurexin functionality are underscored by their binding capability to various postsynaptic ligands, including neuroligins, cerebellins, and leucine-rich repeat transmembrane proteins (LRRTMs) (Francks, 2011; Asede et al., 2020). Such interactions underscore the multifaceted nature of neurexins in synaptic operations and their potential association with neurodevelopmental and neuropsychiatric disorders when dysfunctional (Kasem et al., 2018). Perturbations in neurexin signaling have been implicated in various neurodevelopmental and neuropsychiatric disorders, including autism spectrum disorders (ASD), schizophrenia (SCZ), bipolar disorder (BD), and attention deficit hyperactivity disorder (ADHD) (Kasem et al., 2018; Cuttler et al., 2021).

It should be noted that deletions of NRXN 1–3 and these neurodevelopmental and neuropsychiatric disorders are highly interconnected and not simple one-to-one relationships (Kasem et al., 2018). While not all individuals with NRXN deletions inevitably develop neurodevelopmental or neuropsychiatric disorders, a single NRXN deletion could potentially enhance the risk for a multitude of such disorders. Deletions of neurexin-1α, for instance, have been linked with a significantly elevated risk for SCZ, ASD, and intellectual disability (Reichelt et al., 2012). In contrast, a single disorder could also result from different deletions of neurexin genes. For example, dysfunctions across all three neurexin genes (i.e., NRXN 1–3) have been identified as contributors to a disrupted balance between excitatory and inhibitory neurotransmission in human ASD (Khoja et al., 2023). These findings further highlight the need to figure out the complex association between neurexins and these disorders.

Previous research has investigated the role of neurexins using both *in vivo* and *in vitro* approaches (Cuttler et al., 2021; Gomez et al., 2021). While genetic studies have greatly expanded our understanding of the involvement of neurexins in these disorders (Tromp et al., 2021), a significant gap remains in the functional analysis of these genetic aberrations. Understanding the precise molecular and cellular outcomes of neurexin dysfunction can pave the way for targeted therapeutic interventions. Herein lies the importance of *in vitro* human models, specifically induced pluripotent stem cells (iPSCs). By reprogramming somatic cells from patients into iPSCs, and subsequently differentiating these cells into neurons, researchers can create patient-specific neural models that recapitulate disease phenotypes (Jusop et al., 2023). This approach allows for the study of neurexin dysfunction in a cellular context that closely mirrors the patient’s genetic and epigenetic background (Lee et al., 2020). Meanwhile, somatic cells from healthy population could be used for comparison (Raab et al., 2014). On the other hand, animal models, particularly rodents, have traditionally served as invaluable tools for dissecting the physiological consequences of neurexin dysfunction (Bryda, 2013; Tromp et al., 2021). They offer insights into behavioral manifestations, neuronal circuitry alterations, and potential compensatory mechanisms that might arise due to the dysfunctional neurexin signaling. By employing both iPSCs and animal models, researchers could achieve a more comprehensive understanding that combines human-specific cellular insights with *in vivo* physiological observations (Rowe and Daley, 2019).

Yet, despite the existing plethora of studies on neurexin dysfunction’s relevance to neurodevelopmental and neuropsychiatric disorders, comprehensive systematic review encompassing both iPSC and animal model perspectives is lacking. This systematic review aims to bridge this knowledge gap by amalgamating existing research, shedding light on the molecular, cellular, physiological, and behavioral impacts of neurexin dysfunction, while highlighting both their commonalities and differences.

## Materials and methods

This systematic review was conducted in accordance with the 2020 guidelines and principles detailed in the Preferred Reporting Items for Systematic Reviews and Meta-Analyses (PRISMA) statement and checklist (Shan et al., 2022). The PICOS (Participants; Interventions; Comparisons; Outcomes; Study designs) are defined in Table 1.

**Table 1:** PICOS.

PICOS	Description
P (Participants)	IPSCs (from patients and healthy individuals) and animal models
I (Interventions)	Neurexin dysfunction
C (Comparisons)	Control group
O (Outcomes)	Synaptic/physiological changes and behavioral findings in animal models; genotypic and cellular phenotypic outcomes in IPSCs
S (Study designs)	*In vitro* and *in vivo*

We mainly performed a systematic search of PubMed and Google Scholar databases, targeting original peer-reviewed research articles published in the last 20 years from 10/05/2003 to 10/05/2023. The search terms included combinations of the following: [(Neurexin) OR (NRXN)) *AND* ((IPSC) OR (Induced Pluripotent Stem Cells) OR (Mouse) OR (Rat) OR (Animal)) *AND* ((Schizophrenia) OR (Autism) OR (Bipolar disorder) OR (Attention-deficit/hyperactivity disorder) OR (ADHD) OR (Neurodevelopmental disorders) OR (Neuropsychiatric Disorders)]. Only English language papers were included. Papers were initially screened by *Title* and *Abstract Only*, and excluded if deemed not relevant because the study was not on animal or iPSCs models, neurexin, or any neurodevelopmental/neuropsychiatric disorder. In a second round of screening, full-text papers were assessed for eligibility, and excluded if not relevant to our objectives. Additional search criterion was studies from the manually screening of the reference lists of extracted articles between 2003 and 2023, in order to identify additional studies that may fit the systematic review objective in the current article but were not identified by the PubMed and Google Scholar databases. In case of circular demonstration of research results combined with original empirical articles, only empirical studies were retained if a systematic or narrative review addressing the potential linkage between neurexin dysfunction and these disorders emerged (Shan et al., 2021).

During the second round of screening, we meticulously screened pertinent studies that utilized animal models or IPSCs as their primary methodologies. Emphasis was placed on retaining studies that elucidated synaptic/physiological activity and/or animal behavioral outcomes pertinent to neurodevelopmental and/or neuropsychiatric disorders as a result of neurexin dysfunction. Conversely, studies that lacked explicit mention of the potential association between neurexin dysfunction and these disorders were excluded.

For the studies employing animal models, the extracted data encompassed the targeted neurexin, type of animal used, generation strategy of the model, model viability, synaptic/physiological activities, principal behavioral findings, and the inferred neurodevelopmental or neuropsychiatric disease correlation. On the other hand, data retrieval from studies employing IPSCs comprised the targeted neurexin, donor details and sample count, IPSC model generation technique, genotypic profiles, cellular phenotypic outcomes, and the extrapolated disease correlation.

In addition, we utilized certain methods to evaluate bias risk in both *in vitro* and *in vivo* studies. The quality and risk for bias in the included *in vitro* (i.e., IPSC) studies were assessed by the adapted quasi-experimental studies appraisal tool by the Joanna Briggs Institute (Tufanaru et al., 2017). Each *in vitro* investigation was designated a risk status: low (where any detected bias would not substantively skew results), unclear (where potential bias introduces some result uncertainty), or high (indicating that bias could significantly compromise findings). For *in vivo* research, we employed the SYRCLE risk assessment tool, which refers to the Systematic Review Centre for Laboratory Animal Experimentation’s risk of bias tool, tailored for the included animal studies (Silveira et al., 2023). This instrument is structured around various bias categories: selection, performance, attrition, reporting, and other biases. The current systematic review involved a ten-question evaluation of the articles, with possible responses being “YES” (signifying minimal bias risk), “NO” (indicative of pronounced bias risk), and “UNCLEAR” (signifying indeterminate bias risk).

## Results

**Figure 1:**
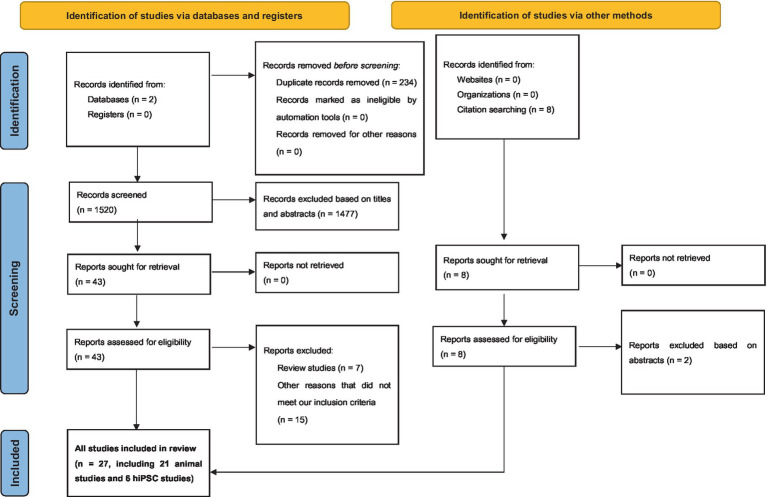
Preferred Reporting Items for Systematic Reviews and Meta-Analyses (PRISMA) diagram demonstrating search strategy.

**Figure 2:**
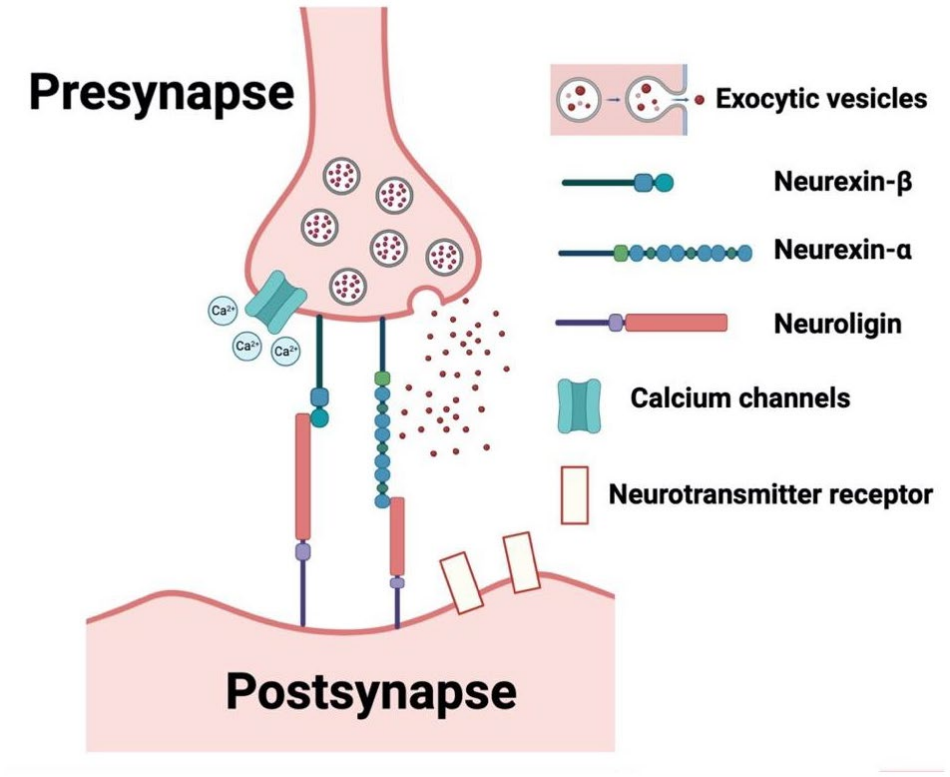
Schematic representation of neurexin-neuroligin interaction. (Created with BioRender.com, accessed on 28 May 2023).

From our search on the PubMed and Google Scholar databases, we procured a total of 1754 records. Subsequent exploration through a ‘citation searching’ methodology yielded an additional 8 records. Firstly, 234 were identified as duplicates and thus removed. Following a preliminary assessment of titles and abstracts, we excluded 1,477 of the 1,528 entries. Subsequently, 51 articles progressed to an advanced screening phase, from which a further 24 were eliminated due to their irrelevance to our stipulated inclusion criteria or their categorization as systematic/narrative reviews upon thorough examination of their full text. Consequently, 27 articles formed the core of our review. Referring to Figure 1 for a detailed overview of our search and screening procedures. All these studies, without exception, probed the potential link between neurexin dysfunction and neurodevelopmental and/or neuropsychiatric disorders, utilizing either animal or IPSC models as their investigative platforms. A schematic representation of neurexin-neuroligin interaction is shown in Figure 2.

**Figure 3:**
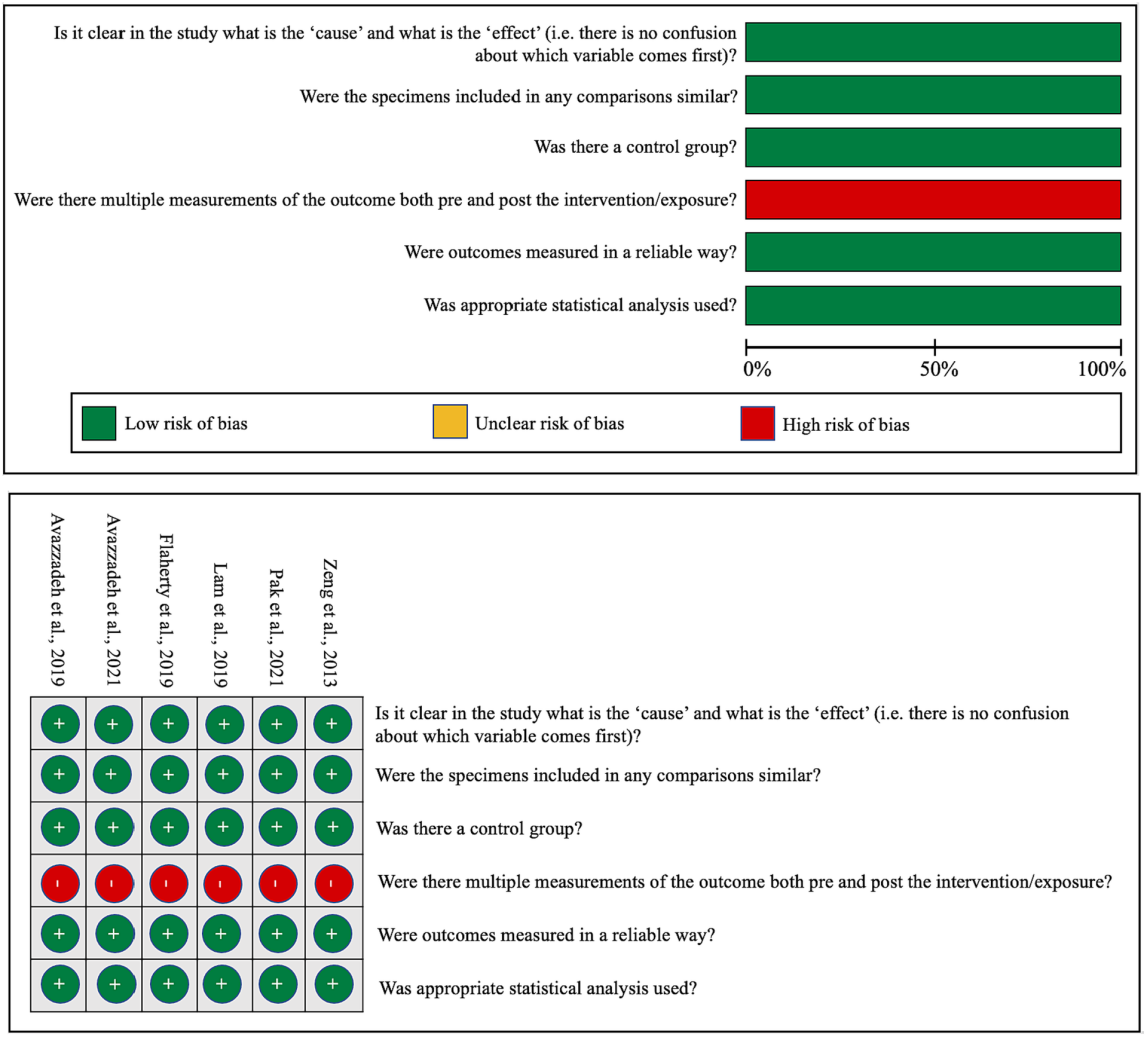
Qualitative analysis with adapted the quasi-experimental studies appraisal tool by the Joanna Briggs Institute.

**Figure 4:**
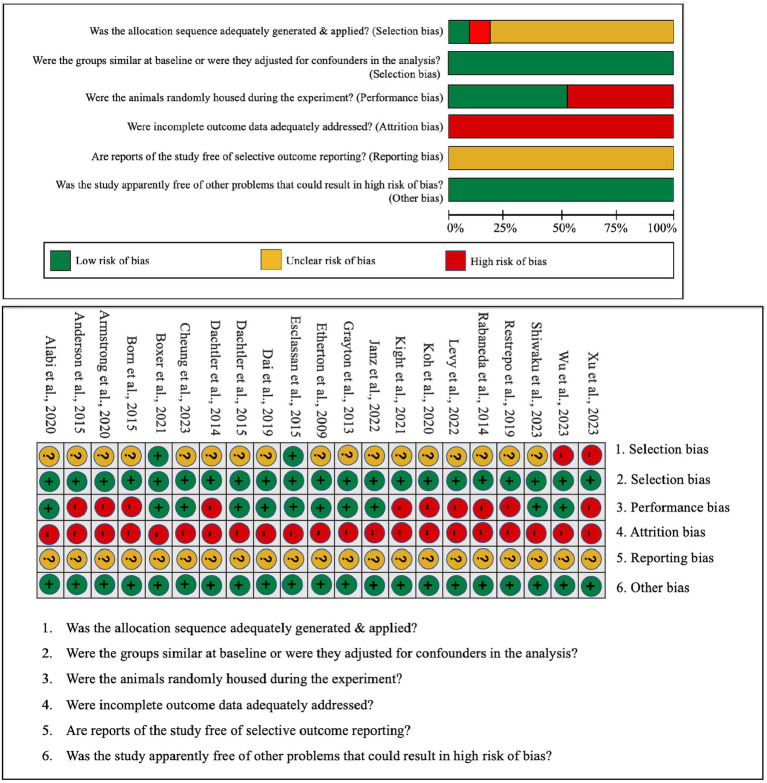
Qualitative analysis with Syrcle’s risk of bias tool for animal studies.

In an effort to gauge the risk of bias in both *in vitro* and *in vivo* studies, we have encapsulated our quality assessment findings in Figure 3 (pertaining to IPSC studies) and Figure 4 (relevant to animal studies). A noteworthy observation is that a predominant fraction of the *in vitro* studies showcased minimal bias risk. However, an anomaly was discerned for the criterion addressing the frequency of outcome measurements both pre and post-intervention/exposure. Herein, a palpable bias risk was evident, attributed largely to these studies’ inclination towards contrasting different cell lines (notably control vs. NRXN-deleted) rather than employing iterative measurements on identical cells prior to and subsequent to specific interventions. As for *in vivo* studies, we identified salient deficiencies in certain quality components. For instance, concerning the criterion, “Was the allocation sequence generated and executed appropriately?” a mere pair of studies (Esclassan et al., 2015; Boxer et al., 2021) demonstrated a minimal bias risk owing to their explicit randomization acknowledgment. Conversely, a sizable cohort exhibited pronounced bias risks due to ambiguous animal selection during result evaluation and/or a lack of randomized housing during experimentation. Furthermore, universal bias risks were detected in the realm of incomplete outcome data reporting. In addition, the absence of accessible study protocols introduced ambiguity in reporting bias, making it challenging to ascertain if the pre-determined primary and secondary outcomes were duly reported. Lastly, when assessed against the criterion, “Was the study apparently free of other problems that could result in high risk of bias?,” all studies uniformly exhibited a minimal bias risk.

The categorization of the 27 key studies according to their research models showed that 21 utilized animal models, while 6 employed IPSC models. An analysis of the neurexin targets within these studies revealed a distinct emphasis on nrxn1/NRXN1, especially Nrxn1α/NRXN1α. Notably, a substantial majority (more than 75%, *n* = 21) of these studies suggested a link to ASD as a potential associated condition, with other neurodevelopmental and neuropsychiatric disorders like schizophrenia, ADHD, and intellectual disability also being mentioned.

Within the 21 studies focusing on animal models, rodents, specifically rats and mice, were predominantly used in 19 of them, while Zebrafish and *Drosophila melanogaster* were each featured in one study. The research heavily favored Nrxn1α, explored in 14 studies, with less frequent examination of Nrxn2α, Nrxn3α, and Nrxnβ. Most studies adopted knockout (KO) strategies in mice, targeting specific Nrxn genes such as Nrxn1α, Nrxnβ, and Nrxn3. Variability in genetic backgrounds of these models (e.g., pure C57BL/6 J, mixed backgrounds) was noted, potentially contributing to phenotypic differences observed. For instance, while Etherton et al. (2009) and Armstrong et al. (2020) both researched Nrxn1α KO mice, their differing genetic backgrounds led to distinct behavioral outcomes, including increased aggression and impaired prepulse inhibition, respectively. Concerning model viability, the majority of studies successfully developed viable models. The earliest Nrxn loss of function mouse models were introduced by Missler et al. (2003) who generated single, double, and triple Nrxn1α/2α/3α KO mice. Tromp et al. (2021) highlighted the importance of maintaining at least two intact α-Nrxns for survival, based on variable survival rates in double and triple KO mice. In a more recent study, Cheung et al. (2023) discovered that triple Nrxn knockout in 5-HT neurons led to a significant reduction in serotonin neurons during early postnatal stages, indicating the viability of these models but with specific neural deficits. The studies consistently reported synaptic and physiological changes due to Nrxn gene disruptions. For example, Alabi et al. (2020) observed neural signaling disruptions in the striatum, and Born et al. (2015) noted a decrease in spontaneous transmitter release at excitatory synapses. Behavioral findings in these animal models displayed both similarities and variances. Commonly reported were deficits in social interactions and increased anxiety-like behavior, aligning with traits in ASD and Schizophrenia, as shown in studies like Dachtler et al. (2014) and Born et al. (2015). However, discrepancies were also apparent; Grayton et al. (2013) reported reduced locomotor activity and increased aggression, while Etherton et al. (2009) observed normal social and anxiety-like behaviors and locomotor activity. These variations suggest that the behavioral impacts of Nrxn disruptions can significantly differ due to the genetic background of the mice. The primary diseases correlated with these disruptions are predominantly ASD and schizophrenia, reflecting the strong link between Nrxn gene disruptions and these disorders. Nonetheless, the observed range of behavioral phenotypes, from social deficits to cognitive impairments, implies that the influence of these genes extends beyond these disorders, potentially affecting a wider spectrum of neurodevelopmental conditions.

In the hiPSC studies analyzed, hiPSC lines originated from diverse sources such as skin biopsies and peripheral blood mononuclear cells, collected from both healthy subjects and patients with disorders including ASD and schizophrenia. While the generation of these iPSC models consistently utilized cellular reprogramming techniques, there was a slight variation in the genetic focus across different studies. The primary attention was on NRXN1α, with investigations into the impacts of NRXN1α+/− deletions, exemplified by Avazzadeh et al. (2019, 2021), and bi-allelic NRXN1-alpha deletions, as reported by Lam et al. (2019). These studies primarily explored intragenic deletions affecting NRXN1, uncovering a spectrum of cellular phenotypes indicative of impaired neuronal function, such as the altered dynamics of calcium and sodium and modifications in neurotransmitter release, as detailed by Avazzadeh et al. (2019, 2021) and Pak et al. (2021). The cellular phenotypes identified exhibited both commonalities and differences; for instance, Avazzadeh et al. (2021) and Pak et al. (2021) observed changes in ion dynamics and neurotransmitter release, whereas Lam et al. (2019) noted a shift in cells towards a radial glia-like identity. Predominantly, the genetic and cellular alterations were linked to ASD and schizophrenia. Nevertheless, the observed range of symptoms and associated conditions, such as seizures, intellectual disability, and developmental delays, indicate that the influence of these genetic modifications might extend well beyond these disorders. This broader impact is exemplified by Avazzadeh et al. (2021), who associated NRXN1α+/− deletions with ASD and related symptoms, and Flaherty et al. (2019), who connected NRXN1 deletions to schizophrenia and bipolar disorder with psychosis.

## Discussion and future directions

Our review systematically examines Nrxn knockout/knock-in (KO/KI) animal models and human-induced pluripotent stem cell (hiPSC) studies on neurexins, highlighting their complex roles in various neurodevelopmental and neuropsychiatric conditions. Utilizing diverse methods, from CRISPR/Cas9 genome editing to hiPSC-derived neuronal cultures, these studies showcase the significant effects of neurexin variations, notably in disorders like ASD and schizophrenia (Avazzadeh et al., 2019; Flaherty et al., 2019). Neurexin dysfunction is also linked to other conditions such as ADHD, intellectual disability, seizures, and developmental delays. Tables 2, 3 provide in-depth information on the specifics of each hiPSC study and Nrxn KO/KI animal investigation, including target neurexins, the animals used, model generation strategies, model viability, synaptic or physiological activities, key findings regarding animal behaviors, and related diseases.

**Table 2:** Overview of existing Nrxn KO/KI animal models detailing target neurexin, animal use, model generation, model viability, synaptic/physiological activity, main findings regarding animal behaviors, and inferred correlated disease.

References	Target neurexin	Animal use	Model generation	Model viability	Synaptic/physiological activity	Main findings regarding animal behaviors	Inferred correlated disease
Alabi et al. (2020)	Nrxn1α	Mouse	Conditional and constitutive KO mice	One Neurexin1a−/− mouse died in the early stages of training, but the rest were viable after genetic modification.	Disruption of Neurexin1a affected neural signals within the striatum, a critical area for feedback-based reinforcement learning.	Mice with Nrxn1α disruption had difficulties selecting beneficial outcomes and avoiding costlier options due to deficits in value representation and updating.	ASD and Schizophrenia
Anderson et al. (2015)	Nrxnβ	Mouse	Neurexin-β-floxed (NBF) mice were generated by homologous recombination targeting the 5′ unique exon for each of the three β-neurexin genes	Viable but smaller and infertile in β-neurexin deficient mice	β-Neurexin KO impairs action-potential induced Ca2 + −influx into presynaptic terminals and enhances basal endocannabinoid activity	Conditional knockout of β-neurexins in CA1-region neurons impaired contextual fear memories	ASD and Schizophrenia
Armstrong et al. (2020)	Nrxn1α	Mouse	Homozygous KO in pure C57BL/6 J background.	The Nrxn1α KO mice were viable but showed physiological variations including reduced body temperature, decreased weight, and altered righting reflex latencies.	N/A	increased levels of social deficits and aggression	ASD
Born et al. (2015)	Nrxn2α	Mouse	Generated and subjected to 8 generations of additional backcrossing to transfer the knockout allele onto a C57BL/6 J genetic background.	N/A	reduced spontaneous transmitter release at excitatory synapses in the neocortex	Mice lacking Nrxn2α exhibited behavioral abnormalities, characterized by social interaction deficits and increased anxiety-like behavior	ASD and Schizophrenia
Boxer et al. (2021)	Nrxn3	Mouse	bred at the University of Colorado Anschutz and had a mixed genetic background of B6;129 or B6.Cg	viable and underwent experiments at P35–42 in visibly healthy conditions	Nrxn3 knockout results in an impairment of synapse density, reduced postsynaptic strength, and diminished IPSC amplitude at PV-RS synapses in males, while the knockout enhances presynaptic release and increases IPSC amplitude in females	N/A	Anxiety
Cheung et al. (2023)	Nrxn1,2,3	Mouse	Creating 5-HT neuron-specific triple Nrxn knockout mice by crossing Fev/RFP mice with the Nrxn1f/f/2 f/f/3 f/f mouse line on a C57BL/6 J background	Mice with Nrxns disrupted in 5-HT neurons were viable but exhibited a decreased number of serotonin neurons in the early postnatal stage.	Impaired 5-HT release, decreased 5-HT release sites and serotonin transporter expression.	Reduced sociability and increased depressive-like behavior	Depression
Dachtler et al. (2014)	Nrxn2α	Mouse	B6;129-Nrxn3tm1Sud/Nrxn1tm1Sud/Nrxn2tm1Sud/J mice were initially obtained and were subsequently outbred to the C57BL/6NCrl strain, with resultant mice being Nrxn2α KO heterozygotes but wild-type for Nrxn1 and Nrxn3	Nrxn2α KO mice were viable and were assessed behaviorally	Real-time PCR analysis showed significant decreases in mRNA levels of genes encoding proteins related to both excitatory and inhibitory transmission, and Munc18-1 protein levels were significantly decreased in the hippocampus of Nrxn2α KO mice, indicating potential deficiencies in presynaptic vesicular release	Nrxn2α KO mice exhibited deficits in sociability and social memory, an anxiety-like phenotype, no preference for new vs. known conspecifics, and similar exploration times for soiled and clean bedding	ASD
Dachtler et al. (2015)	Nrxn1α & Nrxn2α	Mouse	Commercially generated and validated heterozygous KO mice.	N/A	Hippocampal Munc-18 expression remained unchanged, as compared to compared to WT mice.	Nrxn1α HET mice exhibit slight cognitive impairments, whereas Nrxn2α HET mice display significant sociability and social recognition deficits.	ASD and Schizophrenia
Dai et al. (2019)	Nrxn1 & Nrxn2	Mouse	Conditional KI mice for Nrxn1 and Nrxn2 were generated with their genes modified for alternative splicing, and all mice had a mixed hybrid genetic background containing Sv129, C57/Bl6, and CD1 components.	Viable and fertile	N/A	Contextual memory as measured in hippocampal CA1 region	ASD and Schizophrenia
Esclassan et al. (2015)	Nrxn1α	Rat	generated using rats with a biallelic deletion of the Nrxn1-gene on a Sprague Dawley background	Viable	N/A	Nrxn1α-KO rats exhibited hyperactivity, deficits in simple instrumental and spatial-dependent learning, and impaired latent inhibition with an exaggerated startle response.	ASD
Etherton et al. (2009)	Nrxn1α	Mouse	derived from matings between heterozygous neurexin-1α KO mice	viable and suitable for behavioral and electrophysiological studies	reduced spontaneous excitatory synaptic transmission and decreased evoked excitatory synaptic strength in the CA1 region of the hippocampus	impaired prepulse inhibition, increased grooming behaviors, impaired nest-building activities, normal social behaviors, normal anxiety-like behaviors and locomotor activity, and enhanced motor learning on the rotarod.	ASD and Schizophrenia
Grayton et al. (2013)	Nrxn1a	Mouse	Generated by knocking out Nrxn1a on a C57BL6/SV129 mixed genetic background	The homozygous Nrxn1a KO mice were viable and capable of being tested behaviorally	N/A	Homozygous Nrxn1a KO mice showed altered social approach, reduced social investigation, reduced locomotor activity, and male KO mice displayed increased aggression	ASD and Schizophrenia
Janz et al. (2022)	Nrxn1α	Rat	Nrxn1α−/− rats and wildtype littermates were used	Viable	pronounced increases in spontaneous gamma oscillatory power and alterations in auditory-evoked oscillations	Nrxn1α−/− rats displayed locomotor hyperactivity, increased moving time and distance, and altered auditory-evoked responses, but their responses to social stimuli remained intact	ASD and Schizophrenia
Kight et al. (2021)	Nrxn1α	Rat	Commercially generated and validated heterozygous KO mice.	N/A	N/A	Increased locomotive behavior in male rats.	Neurodevelopmental disorders (e.g., ASD and schizophrenia)
Koh et al. (2020)	Nrxn2αα	Zebrafish	The nrxn2aa−/− mutants were generated using the CRISPR/Cas9 method	Homozygous mutant embryos from heterozygous parents showed no overt defects, but maternal-zygotic (MZ) nrxn2aa−/− mutants displayed branched axons and defective motor neurons, while zygotic mutants developed normally	impaired synapse formation	increased anxiety	ASD
Levy et al. (2022)	Nrxn1	*Drosophila melanogaster*	Generated using fly strains containing the Nrx-1273 and Nrx-1241 alleles	Nrx-1-null flies were viable but showed decreased resistance to nutrient deprivation, heat stress, and impaired flight ability.	Nrx-1 mutants showed significantly decreased glycogen and NAD + levels.	Nrx-1 mutants showed seizure-like behavior after mechanical stimulation	Seizure
Rabaneda et al. (2014)	Nrxn1β	Mouse	Generated using the HA-nrxn1β△C construct	Viable	Impairment of glutamatergic synaptic transmission	increased self-grooming, deficits in social interactions, and altered interactions for nonsocial olfactory cues	ASD
Restrepo et al. (2019)	Nrxn3α	Mouse	Developed using mouse hippocampal cultures, *in vivo* stereotactic injections, and *ex vivo* brain slices.	Viable at various stages post-birth and post-injection	The A687T mutation in neurexin-3α enhanced presynaptic morphology and increased presynaptic neurotransmitter release at excitatory synapses.	N/A	intellectual disability and epilepsy
Shiwaku et al. (2023)	Nrxn1α	Mouse	Mice were exposed to anti-NRXN1α autoantibodies isolated from patients with schizophrenia to generate the model	remained viable after antibody administration	reduced frequency of the miniature excitatory postsynaptic current in the mice’s frontal cortex.	Mice treated with the autoantibody displayed reduced cognition, impaired pre-pulse inhibition, and decreased social novelty preference.	Schizophrenia
Wu et al. (2023)	Nrxn1α	Rat	Male Sprague Dawley rats were subjected to PFC knockdown of NRXN1 using intracerebral injection of AAV9-NRXN1-GFP	After intracerebral injections, the animals were monitored daily and given 2 weeks for full expression of the viral constructs before behavioral testing	Nrxn1 downregulation in the medial PFC induced impaired neurite outgrowth in prefrontal neurons	Increased anxiety-like behaviors and abnormal social phenotypes.	Neurodevelopmental disorders (e.g., ASD and ADHD)
Xu et al. (2023)	Nrxn1α	Mouse	Homozygous and heterozygous deletions (ΔExon9 and ΔIntron17) in the C57BL/6 J background using both traditional crossing techniques and CRISPR/Cas9-mediated genomic editing	N/A	N/A	Nrxn1 mutant mice showed no significant alterations in anxiety or locomotion, but exhibited impairments in social interactions and disturbances in circadian rhythms relevant to autism.	ASD

**Table 3:** Summary of hiPSC studies on NRXN outlining details of target neurexin, donor data and sample count, IPSC model generation, genotypes, cellular phenotypes and inferred correlated disease.

References	Target neurexin	Donor Data and Sample Count	IPSC model generation	Genotypes	Cellular phenotypes	Inferred correlated disease
Avazzadeh et al. (2019)	NRXN1	Skin biopsies were taken from five healthy donors and three ASD patients, resulting in seven control and six NRXN1α+/− iPSC lines	iPSCs were derived from dermal fibroblasts and characterized for pluripotency using various markers	The genotypes of interest are the NRXN1α+/− deletions observed in three ASD patients	NRXN1α+/− neurons exhibited altered calcium dynamics, with increased frequency, duration, and amplitude of Ca2+ transients	ASD
Avazzadeh et al. (2021)	NRXN1α	There were five NRXN1α+/− iPSC lines from three ASD patients and six control iPSC lines from five healthy donors.	derived from skin biopsies and were reprogrammed under manufacturer’s instructions.	Three ASD patients carried different NRXN1α+/− deletions affecting various exons.	NRXN1α+/− cortical neurons displayed larger sodium currents, higher AP amplitude, accelerated depolarization time, and had transcriptomic changes with upregulated glutamatergic synapse and ion channels	ASD, with associated symptoms like seizures, intellectual disability, developmental delay, and language delay
Flaherty et al. (2019)	NRXN1	generated from four individuals with rare heterozygous intragenic deletions in NRXN1 diagnosed with psychosis disorders, along with one related and three unrelated age-, sex-, and ethnicity-matched controls.	IPSCs were derived from fibroblasts using a non-integrating sendai virus approach, and further differentiated into neuronal and neural progenitor cell (NPC) types.	Four individuals had heterozygous intragenic deletions in NRXN1: two with ~136-kb deletions in the 3′-region and two with ~115-kb deletions in the 5′-region.	hiPSC-neurons consisted mainly of glutamatergic neurons, but also included GABAergic neurons and astrocytes; declined neuronal activity.	Schizophrenia and bipolar disorder with psychosis
Lam et al. (2019)	NRXN1α	IPS cells were generated from an individual diagnosed with ASD carrying bi-allelic NRXN1-alpha deletion and four healthy control individuals (Ctrl-7, Ctrl-3, Ctrl-9, Ctrl-10)	Generated from skin fibroblasts, induced toward neuroepithelial stem (NES) cells, and then differentiated to neurons for various durations	The genotypes investigated include an individual with a bi-allelic NRXN1-alpha deletion and multiple healthy controls	Cells carrying the NRXN1-alpha deletion shifted towards radial glia-like identity with a higher proportion of differentiated astroglia and immature neuronal cells	ASD
Pak et al. (2021)	NRXN1	Peripheral blood mononuclear cell (PBMC) specimens and genomic DNA were obtained from schizophrenia patients carrying heterozygous NRXN1 exonic deletions and control individuals from the Molecular Genetics of Schizophrenia (MGS2) European-ancestry cohort.	Generated from PBMCs by the Rutgers University Cell and DNA Repository (RUCDR) using integration-free Sendai virus reprogramming.	Whole-genome sequencing (WGS) validated the presence of an exonic NRXN1 deletion in patients but not in controls.	Human neurons with heterozygous NRXN1 deletions exhibited impaired neurotransmitter release and increased CASK protein levels, while Nrxn1-deficient mouse neurons did not show the same phenotypes.	ASD and schizophrenia
Zeng et al. (2013)	NRXN1	Human fetal dermal fibroblasts (HDFf) were acquired from ATCC	generated from skin fibroblasts and then differentiated into neural stem cells (NSCs) using a PiggyBac transposon reprogramming system.	The genotypes involve exonic deletions in NRXN1	NRXN1 knockdown resulted in a decline in astrocyte marker GFAP.	ASD, schizophrenia, and developmental delay

Recognizing the genetic link between neurexin variants and an increased risk for neurodevelopmental and neuropsychiatric disorders, our study seeked to understand the neuronal functions of these genes and their potential role in initiating, progressing, and exacerbating mental disorders. We have expanded upon previous reviews by generating animal models of Nrxns and integrating relevant data from hiPSC studies to better evaluate the mental conditions associated with these genes (Tromp et al., 2021).

### Neurexin dysfunction in autism-spectrum disorders

ASD, as a complex neurodevelopmental disorder, presents with a spectrum of cognitive and behavioral phenotypes, including social interaction deficits and repetitive behaviors (Evans et al., 2023). The identification of rare missense mutations in exon 1 of NRXN1β in ASD patients (p.S14L and p.T40S), absent in controls, first established a genetic link with NRXNs (Feng et al., 2006). Recent research has increasingly highlighted the role of NRXN1α variants in the development of ASD (Avazzadeh et al., 2021). The distinct behavioral phenotypes observed in Nrxn1 mutant mice models, such as social deficits and altered circadian rhythms (Levy et al., 2022), resonate with the human ASD condition, suggesting a possible conserved molecular mechanism across species. The resemblance in behavioral phenotypes could be attributed to the fundamental role of NRXN1 in synaptic formation and neurotransmitter release, processes crucial for normal social and cognitive functions.

In hiPSC models derived from ASD patients, perturbations in calcium dynamics and ionic pathways (Avazzadeh et al., 2019) point towards a synaptic dysfunction at a cellular level. Such findings may explain the diverse cognitive and behavioral outcomes in ASD, aligning with theories that posit synaptic homeostasis disruptions as a core component of ASD pathophysiology (Bourgeron, 2015). The variation in cellular responses to NRXN1 mutations among different studies might be attributed to the inherent genetic diversity among individuals with ASD, reflecting the heterogeneity of the disorder. Furthermore, the impact of NRXN1 mutations extends beyond synaptic dysfunction to broader neural network alterations. The observation of a shift towards radial glia-like identity in cells with NRXN1-alpha deletion (Lam et al., 2019) suggests a profound effect on neural development. This could potentially disrupt the balance between excitatory and inhibitory circuits, a crucial aspect of ASD’s neural basis.

While most ASD research has focused on NRXN1 deletions, studies also implicate NRXN2 and NRXN3. While some research, such as Gauthier et al.’s, identified specific truncating mutations in NRXN2α associated with ASD (Gauthier et al., 2011), other studies reported broader *de novo* deletions in NRXN2 linked to autistic behaviors and developmental delays (Mohrmann et al., 2011; Boyle et al., 2015). Additionally, a study by Vaags et al. (2012) found NRXN3 mutations in both individuals with ASD and their unaffected siblings, indicating reduced penetrance and suggesting a complex genetic interaction rather than a straightforward cause-effect relationship. In contrast, a study by Yuan et al. reported a co-segregating NRXN3α deletion in a child with ASD (Yuan et al., 2018). In contrast, a study by Yuan et al. reported a co-segregating NRXN3α deletion in a child with ASD. This evidence indicates an inconsistent role for NRXN2 and NRXN3 in ASD, and further research is needed to clarify their specific contributions to the disorder.

### Neurexin dysfunction in schizophrenia

Neurexin variations, particularly those in NRXN1, are consistently implicated in schizophrenia (Flaherty et al., 2019; Pak et al., 2021), a severe mental disorder characterized by delusions, hallucinations, and cognitive deficits (Iqbal et al., 2023). Studies have established a strong link between NRXN1 and schizophrenia. For example, rodent models exposed to anti-NRXN1α autoantibodies from schizophrenia patients exhibited cognitive impairments typical of the disorder (Restrepo et al., 2019; Shiwaku et al., 2023). Concurrently, hiPSC studies have shown reduced neuronal activity and neurotransmitter release in neurons from schizophrenia patients with NRXN1 deletions (Flaherty et al., 2019; Pak et al., 2021). This aligns with the known role of neurotransmitters like dopamine, glutamate, and serotonin in the pathophysiology of schizophrenia, underscoring neurexins’ relevance in this context (Beeraka et al., 2022).

As of now, there has been no research confirming a connection between NRXN2 and schizophrenia. However, a significant study on the NRXN3 gene and schizophrenia was conducted with a Chinese Han population, involving 1,214 schizophrenia patients and 1,517 control subjects. This study identified three specific Single Nucleotide Polymorphisms (SNPs) within NRXN3 that were associated with schizophrenia, located in the first and second introns of the gene (Hu et al., 2013). These findings suggest a potential link between NRXN3 and schizophrenia, though further research is needed for a more comprehensive understanding.

### Neurexin dysfunction in other neurodevelopmental/neuropsychiatric disorders

The impact of neurexins extends beyond ASD and schizophrenia, affecting a variety of other conditions. Mutations in Nrxn3α, for example, are linked to intellectual disabilities and epilepsy (Shiwaku et al., 2023). Research has also shown that Nrxn1 influenced seizure-like behaviors in flies (Avazzadeh et al., 2019). Additionally, studies indicated that individuals with epilepsy were more likely to develop certain neuropsychiatric conditions (Tolchin et al., 2020). Further, it has been found that reduced Nrxn1 expression in rats could lead to symptoms associated with various neurodevelopmental disorders, including ADHD (Wu et al., 2023). These varied symptoms demonstrate the wide-ranging role of neurexins in synaptic functions, suggesting that disruptions in neurexin activity can trigger a range of neurodevelopmental and neuropsychiatric symptoms, influenced by other genetic and environmental factors (Gupta et al., 2023). The overlapping symptoms across different disorders point to potential common molecular mechanisms that warrant further investigation.

### Advantages and limitations

Our review’s expansive scope, encompassing both animal and human-derived model systems from fruit flies to humans, provides a holistic perspective on neurexin’s neurobiological significance. While animal models shed light on multifaceted behaviors, hiPSC models offer unparalleled cellular-level details, bridging the translational gap (Xu et al., 2023).

Nevertheless, challenges persist. A significant limitation across the studies is the diversity in methodologies, ranging from different techniques in model generation, such as traditional crossing vs. CRISPR/Cas9 genomic editing, to various animal models and iPSC sources (study methodologies vary, introducing potential inconsistencies). The pronounced focus on ASD and schizophrenia might inadvertently minimize neurexins’ role in other neurodevelopmental or neuropsychiatric disorders. While animal models are informative, translating these findings directly to humans can be uncertain. Additionally, hiPSC models, despite their sophistication, may not fully capture the intricacies of human brain development *in vivo* (Dixon and Muotri, 2023).

Our review primarily concentrates on certain models and does not include others, such as human pluripotent stem cell-derived forebrain organoid models. These models, as discussed in the study by Sebastian et al. (2023), provided essential insights developmental-timing- and cell-type-specific vulnerabilities associated with NRXN1 deletions in the context of schizophrenia. Omitting these advanced models might lead to a gap in our understanding of neuronal development and the pathology related to NRXN1 gene changes. Furthermore, the current research landscape is heavily focused on NRXN1, with less attention given to the roles of NRXN2 and NRXN3.

### Future recommendations

To the best of our knowledge, this is the first systematic review study to present potential associations between neurexin dysfunction and a spectrum of neurodevelopmental and neuropsychiatric disorders. Moving forward, several key research considerations must be addressed. First, we believe there is an undeniable urgency for establishing standardized protocols in generating animal and hiPSC models, in order to provide a consistent framework for comparison and replication. Second, while the current literature predominantly focuses on NRXN1, future endeavors should cast a wider net to thoroughly investigate NRXN2 and NRXN3. Thirdly, future studies would benefit from incorporating data from organoid models, as discussed earlier, to gain a more complete understanding of NRXN-related disorders. Lastly, there is a notable imbalance between studies using animal models and those employing hiPSCs. Given hiPSCs’ ability to provide unique insights into human-specific cellular processes, increasing the number of hiPSC studies with varied sample sizes is essential. These studies not only enhance our understanding of human cellular and molecular dynamics but also pave the way for personalized medicine through the use of patient-derived cells.

## Conclusion

This systematic review comprehensively explores the connections between neurexin dysfunction and a range of neurodevelopmental and neuropsychiatric disorders, employing a dual approach of animal models and hiPSC studies. The review highlights the significant implications of neurexin dysregulation, particularly in ASD and schizophrenia, while also acknowledging the broad spectrum of other neurodevelopmental disorders influenced by neurexin anomalies. The findings emphasize the need for more standardized methodologies in future research, particularly in the development of animal and hiPSC models. Additionally, the review underscores the importance of broadening our investigative focus beyond the primarily studied NRXN1 to include other less explored neurexin variants. This approach is crucial for deepening our understanding of these complex disorders and advancing towards personalized therapeutic interventions. The potential of hiPSC models as powerful tools in this research domain is particularly noted, promising to bridge existing gaps and propel translational research forward.

## Data availability statement

The original contributions presented in the study are included in the article/supplementary material, further inquiries can be directed to the corresponding authors.

## Author contributions

DS: Conceptualization, Methodology, Resources, Validation, Writing – original draft, Writing – review & editing. YS: Conceptualization, Methodology, Writing – original draft, Writing – review & editing. YZ: Conceptualization, Methodology, Data curation, Writing – original draft, Writing – review & editing. CH: Investigation, Methodology, Validation, Visualization, Writing – review & editing. WX: Formal analysis, Methodology, Validation, Visualization, Writing – review & editing. ZL: Investigation, Validation, Visualization, Writing – review & editing. FG: Conceptualization, Investigation, Visualization, Writing – review & editing. QO: Formal analysis, Investigation, Visualization, Writing – review & editing. ZiD: Data curation, Investigation, Methodology, Writing – review & editing. ZhD: Conceptualization, Investigation, Methodology, Resources, Validation, Writing – review & editing.
